# Single-Target Versus Multi-Target Drugs Versus Combinations of Drugs With Multiple Targets: Preclinical and Clinical Evidence for the Treatment or Prevention of Epilepsy

**DOI:** 10.3389/fphar.2021.730257

**Published:** 2021-10-27

**Authors:** Wolfgang Löscher

**Affiliations:** Department of Pharmacology, Toxicology, and Pharmacy, University of Veterinary Medicine Hannover, Germany, and Center for Systems Neuroscience Hannover, Hannover, Germany

**Keywords:** antiseizure drugs, antiepileptic drugs, polypharmacy, designed multiple ligands, drug resistance, epileptogenesis

## Abstract

Rationally designed multi-target drugs (also termed multimodal drugs, network therapeutics, or designed multiple ligands) have emerged as an attractive drug discovery paradigm in the last 10–20 years, as potential therapeutic solutions for diseases of complex etiology and diseases with significant drug-resistance problems. Such agents that modulate multiple targets simultaneously are developed with the aim of enhancing efficacy or improving safety relative to drugs that address only a single target or to combinations of single-target drugs. Although this strategy has been proposed for epilepsy therapy >25 years ago, to my knowledge, only one antiseizure medication (ASM), padsevonil, has been intentionally developed as a single molecular entity that could target two different mechanisms. This novel drug exhibited promising effects in numerous preclinical models of difficult-to-treat seizures. However, in a recent randomized placebo-controlled phase IIb add-on trial in treatment-resistant focal epilepsy patients, padsevonil did not separate from placebo in its primary endpoints. At about the same time, a novel ASM, cenobamate, exhibited efficacy in several randomized controlled trials in such patients that far surpassed the efficacy of any other of the newer ASMs. Yet, cenobamate was discovered purely by phenotype-based screening and its presumed dual mechanism of action was only described recently. In this review, I will survey the efficacy of single-target vs. multi-target drugs vs. combinations of drugs with multiple targets in the treatment and prevention of epilepsy. Most clinically approved ASMs already act at multiple targets, but it will be important to identify and validate new target combinations that are more effective in drug-resistant epilepsy and eventually may prevent the development or progression of epilepsy.

## Introduction

Epilepsy is a common, chronic brain disorder characterized by spontaneous recurrent seizures (SRS) and, often, comorbidities such as anxiety, depression, and cognitive decline (Devinsky et al., 2018). Epilepsy, or the epilepsies, are complex syndromes due to their multi-factorial origins and manifestations (Savage, 2014). There are more than a dozen types of epilepsy and numerous types of epileptic seizures, underlining the complexity of the disease (Savage, 2014). The first-line treatment for epilepsy is antiseizure medications (ASMs; also termed antiepileptic drugs), which symptomatically suppress SRS (Löscher and Klein, 2021a). However, despite the availability of >30 ASMs, about one-third of epilepsy patients are resistant to treatment and this figure has not changed over recent decades (Chen et al., 2018; Janmohamed et al., 2020). Since patients with the same type of clinical seizure may differentially respond to ASMs, the pathophysiological events that underlie epileptic seizures apparently not only differ between unique seizure syndromes, but are also multifactorial for the same type (Löscher and Schmidt, 1994). Thus, 27 years ago, Löscher and Schmidt (1994) wrote that “in order to achieve improved therapy of epilepsy, the real challenge for the future will be to create novel broadly acting antiepileptic drugs with multiple mechanisms of action.” This notion was based on the fact that drug developers traditionally aim towards more and more selective targets, although an absolute selectivity in a drug may in fact not be desirable for complex, multifactorial diseases such as epilepsy (Löscher and Schmidt, 1994). In fact, for various other complex brain diseases, the development of rationally designed multi-target drugs (also termed multimodal drugs or designed multiple ligands [DMLs]) has become an attractive strategy within the pharmaceutical industry (Talevi, 2015; Bain et al., 2017; Lin et al., 2017; Ramsay et al., 2018; Benek et al., 2020; Makhoba et al., 2020). An analysis of the US Food and Drug Administration (FDA)-approved new chemical entities (NCEs) from 2015 to 2017 showed that 21% of all NCEs were DMLs, compared to 34% single-target drugs (Ramsay et al., 2018). Compared to combination therapies, DMLs are thought to present several advantages, including more predictable pharmacokinetics, lower probabilities of drug interactions, and higher patient compliance (Talevi, 2015). However, despite these considerations, only one ASM, padsevonil, has been intentionally developed as a single molecular entity that could target two different mechanisms (Wood et al., 2020). In this review, I will discuss the efficacy of single-target vs multi-target drugs vs multi-target drug combinations in the treatment of epilepsy. Furthermore, I will shortly review the potential efficacy of such strategies for the prevention of epilepsy in patients at risk.

## Preclinical Discovery and Development of Antiseizure Medications

During preclinical development, investigational compounds are typically being tested in a battery of animal models of seizures and epilepsy (Swinyard and Kupferberg, 1985; Löscher and Schmidt, 1988; Bialer and White, 2010; Löscher, 2017; Wilcox et al., 2020). Only compounds that exert antiseizure activity at doses far below those inducing behavioral adverse effects such as sedation or ataxia are developed further. A typical battery of rodent seizure models is shown in Table 1, including the maximal electroshock seizure (MES) test for identifying efficacy against generalized tonic-clonic seizures, the s.c. pentylenetetrazole (PTZ) seizure test for identifying efficacy against nonconvulsive (absence, myoclonic) seizures, and the 6-Hz test for identifying efficacy against difficult-to-treat focal seizures. For the 6-Hz test, different currents (22, 32, or 44 mA) are used for transcorneal induction of focal seizures, which allows differentiation of compounds as shown in Table 1. The higher the current strength, the more resistant is the model to most ASMs (Barton et al., 2001; Metcalf et al., 2017; Wilcox et al., 2020). The MES, PTZ, and 6-Hz models are performed in healthy (non-epileptic) mice or rats by electrical or chemical induction of acute seizures. However, epilepsy is a chronic disease, so models reflecting chronic epileptogenic brain alterations are included in preclinical drug evaluation (Löscher, 2016; Barker-Haliski et al., 2017; Wilcox et al., 2020). The two commonly used chronic models shown in Table 1 are the intrahippocampal kainate mouse model of mesial temporal lobe epilepsy (mTLE), in which electrographic and electroclinical SRS develop after induction of a limbic status epilepticus (SE) induced by unilateral injection of kainate into the hippocampus (Duveau and Roucard, 2017), and the kindling model, in which repeated intermittent electrical stimulation of amygdala or hippocampus lead to an enhanced convulsive response to the initially subconvulsive stimulus (Sato et al., 1990). In addition to electrical kindling via depth electrodes in limbic brain regions, corneal kindling in mice or rats is used as a chronic model of TLE (Wilcox et al., 2020).

**TABLE 1:** Antiseizure potencies of multi-target antiseizure medications (ASMs) in mouse and rat models. Some ASMs that are thought to act predominantly at one target are shown for comparison. Data are from Gladding et al. (1985); Löscher (1980), Löscher et al. (1986); Löscher and Nolting (1991), Löscher and Hönack (1993); Dalby and Nielsen (1997); Otsuki et al. (1998), Barton et al. (2001), Riban et al. (2002); Stöhr et al. (2007); Hanada et al. (2011), Bankstahl et al. (2013), Twele et al. (2015); Duveau et al. (2016); Klitgaard et al. (2016); Wu et al. (2019), Leclercq et al. (2020), Löscher et al. (2021), and the PANAChE database of the NINDS. Abbreviations: i.h., intrahippocampal; MES, maximal electroshock seizure; NE, not effective at tolerated doses; p.o., orally; PTZ, pentylenetetrazole; SRS, spontaneous recurrent seizures; SV, synaptic vesicle protein.

Compound	Targets	ED50 (mg/kg i.p.) at time of peak effect								
		MES		s.c. PTZ		6-Hz (mice)^a^			SRS in i.h kainate model (mice)	Amygdala or hippocampal kindled seizures (rats)^b^
	
		Mice	Rats	Mice	Rats	22 mA	32 mA	44 mA		
**Multi-target ASMs**										
Padsevonil^c^	SV2A,B,C and GABA_A_ receptors	92.8		4.8				0.16	∼0.5	2.43
Cenobamate	GABA_A_ receptors and persistent Na^+^ currents	9.8	2.9	28.5		11	17.9	16.5		16.4
Felbamate	GABA_A_ and NMDA receptors, transient Na^+^ currents, voltage-gated Ca^2+^ channels	35.5	35	126	>250 (p.o.)	13.1	69.5	241		296
Retigabine (ezogabine)	Voltage-gated K^+^ (KCNQ) channels, GABA_A_ receptors	9.3	5.1	13.5			26	33		3.2
Valproate	GABA synthesis, NMDA receptors, persistent Na^+^ currents, low-voltage activated T-type Ca^2+^ channels	271	140	149	195	41.5	126	310	∼330	190
Topiramate	GABA_A_ and NMDA receptors, transient and persistent Na^+^ currents	33	11.5	NE			NE			13.3
Phenobarbital	GABA_A_ and AMPA receptors, voltage-gated Ca^2+^ channels	21.8	12	13.2	41		14.8	18.3	25	16
**Single-target ASMs**										
Phenytoin	Voltage-activated Na^+^ channels	9.5	13	NE	NE	9.4	NE	NE	NE	30
Carbamazepine	Voltage-activated Na^+^ channels	8.8	6	NE	NE		47.9		NE	8
Lamotrigine	Voltage-activated Na^+^ channels	7.5	4,4	NE		4.4	NE	NE	NE	∼3.4
Lacosamide	Voltage-activated Na^+^ channels	4.5	3.9 (p.o.)	NE	NE		9.9			13.5
Brivaracetam	SV2A	113		30				4.4		∼80
Levetiracetam^d^	SV2A	NE		NE		4.6	19.4	1,089	420	∼54
Ethosuximide	Low-voltage activated T-type Ca^2+^ channels	NE	NE	130	140	87	167	NE		NE
Vigabatrin^e^	GABA metabolism	NE		940			>250		50	600
Perampanel (p.o.)	AMPA receptors	1.6		0.94			2.1	2.8	0.7	∼10

a Potency varies with mouse strain used.

b For generalized convulsive seizures (ED_50_s are higher for focal seizures).

c Not approved for treatment of epilepsy.

d Note that levetiracetam also acts via other targets (see text).

e Much more potent after chronic administration.

The advantage of using a battery of animal models as shown in Table 1 is their translational value, which is superior compared to various other areas of neurology (Löscher et al., 2013). Thus, starting with phenytoin in the 1930s, all ASMs were discovered by testing in animal models, such as MES or kindling. The best predictivity of clinical drug activity is obtained by amygdala kindled rats, which correctly predicted the efficacy of numerous ASMs against focal-onset seizures in patients (Table 1). Importantly, testing of novel compounds in the kindling model was more predictive of clinical efficacy than testing in the MES test, as for instance demonstrated by vigabatrin, levetiracetam, and tiagabine (Table 1). The finding of Löscher’s group that levetiracetam is particularly effective in the amygdala kindling model (Löscher and Hönack, 1993) was essential in further developing this compound, which is now one of the most widely used ASMs (Löscher et al., 2016).

As shown in Table 1, ASMs differ markedly in their efficacy in animal models. ASMs can be grouped into three categories: 1) ASMs with a narrow spectrum of preclinical efficacy such as ethosuximide (only active against absence and myoclonic seizures); 2) ASMs which mainly act in MES and focal-onset seizure models (the vast majority of compounds shown in Tables 1 and 2) ASMs with a broad spectrum of efficacies such as brivaracetam, valproate, and alkyl-carbamates, such as cenobamate. At least in part, the preclinical profile of antiseizure efficacy resembles the clinical spectrum (Löscher and Klein, 2021a). For instance, ethosuximide is almost exclusively used for the treatment of absence seizures in humans; phenytoin and carbamazepine act mainly against focal-onset and primary or secondary generalized tonic-clonic seizures in animal models and patients, and valproate exhibits a broad spectrum of preclinical and clinical efficacy.

**TABLE 2:** Perceived molecular targets of clinically used antiseizure medications. Adapted from Rogawski and Löscher (2004), Rogawski et al. (2016), Sills and Rogawski (2020), and Löscher and Klein (2021a).

Mechanistic classes of antiseizure medications		Antiseizure medications that belong to this mechanistic class
*Modulators of voltage-gated sodium channels*		
	Increase of fast inactivation (transient sodium current; I_NaT_)	Phenytoin, fosphenytoin,^a^ carbamazepine, oxcarbazepine,^b^ eslicarbazepine acetate,^c^ lamotrigine; possibly topiramate, zonisamide, rufinamide, brivaracetam
	Increase of slow inactivation	Lacosamide
	Block of persistent sodium currents (I_NaP_)	Cenobamate, lacosamide, carbamazepine, oxcarbazepine, eslicarbazepine, lamotrigine, phenytoin, topiramate, valproate, gabapentin, cannabidiol
*Blockers of voltage-gated calcium channels (T-type)*		
	High-voltage activated (HVA)	Phenobarbital, phenytoin, levetiracetam
	Low-voltage activated T-type (Ca_v_3)	Ethosuximide (Ca_v_3.2 > Ca_v_3.1), methsuximide, eslicarbazepine (Ca_v_3.2), possibly valproate
*Activators of voltage-gated potassium channels* (*K*_*v*_ *7*)		Retigabine (ezogabine)
*Modulators of GABA-mediated inhibition*		
	Allosteric modulators of GABA_A_ receptors	Phenobarbital, primidone, stiripentol, benzodiazepines, (including clonazepam, clobazam, diazepam, lorazepam, and midazolam), topiramate, felbamate, retigabine (ezogabine), cenobamate
	Inhibitors of GAT1 GABA transporter	Tiagabine
	Inhibitors of GABA transaminase (GABA-T)	Vigabatrin
	Activators of Glutamic acid decarboxylase (GAD)	Possibly valproate, gabapentin, pregabalin
*Inhibitors of ionotropic glutamate receptors*		
	Antagonists of NMDA receptors	Felbamate, topiramate, possibly valproate
	Antagonists of AMPA receptors	Perampanel, phenobarbital, levetiracetam, topiramate
*Modulators of the presynaptic release machinery*		
	SV2A	Levetiracetam, brivaracetam
	α2δ subunit of calcium channels	Gabapentin, pregabalin
*Inhibitors of carbonic anhydratase*		Acetazolamide, sulthiame, topiramate, zonisamide, possibly lacosamide
*Serotonin-releasing agents*		Fenfluramine
*Disease-specific modulators*		
	Inhibitors of mTORC1 signaling^d^	Everolimus
	Lysosomal enzyme replacement^e^	Cerliponase alfa (recombinant tripeptidyl peptidase 1)
*Mixed/unknown*		Valproate, felbamate, topiramate, zonisamide, rufinamide, adrenocorticotrophin (ACTH), cannabidiol, cenobamate, potassium bromide

a Fosphenytoin is a prodrug for phenytoin.

b Oxcarbazepine serves largely as a prodrug for licarbazepine, mainly S-licarbazepine (eslicarbazepine).

c Eslicarbarbazepine acetate is a prodrug for S-licarbazepine (eslicarbazepine).

d In patients with epilepsy due to tuberous sclerosis complex (TSC).

e In patients with epilepsy due to neuronal ceroid lipofuscinosis type 2 (CLN2).

In addition to the preclinical models illustrated in Table 1, specific genetic animal models are used as models for generalized absence (spike-wave) seizures such as the GAERS (Genetic Absence Epilepsy Rat from Strasbourg) model and the WAG/Rij (Wistar Albino Glaxo from Rijswijk) rat model (Pearce et al., 2014). Furthermore, specific genetic animal models for pediatric genetic epilepsies, such as Lennox-Gastaut syndrome, infantile spasms (West syndrome), Dravet syndrome, and tuberous sclerosis complex (TSC) can be used to discover novel ASMs for the difficult-to-treat seizures in these syndromes (Demarest and Brooks-Kayal, 2018). Several newer ASMs, including cannabidiol, rufinamide, stiripentol, everolimus, and fenfluramine are almost exclusively used in such genetic epilepsies. Current preclinical models of pediatric epilepsies include mouse, rat, and zebrafish models carrying the mutations that are responsible for the genetic epilepsies. Furthermore, *in vitro* models, such as induced pluripotent stem cells (iPSCs), are increasingly used for screening novel compounds for the treatment of epileptic encephalopathies (Niu and Parent, 2020). iPSCs are considered a more translational model than the usual *in vitro* cellular or slice models employed, but the drug effects observed in these cells are not considered as a preclinical evidence from the regulatory agencies.

In the last ∼45 years, the development of ASMs was spurred largely by the Epilepsy Therapy Screening Program (ETSP; known until 2017 as the Anticonvulsant Screening Program (ASP)), set up in 1975 by J. Kiffin Penry at the National Institutes of Neurological Disorders and Stroke (NINDS) of the U.S. National Institute of Health (Porter and Kupferberg, 2017). One of the major aims of the ETSP is to identify drugs with efficacy against drug-resistant seizures. As previously described in detail (Kehne et al., 2017; Wilcox et al., 2020), preclinical drug testing in the ETSP is divided into an initial “identification” phase, followed by a “differentiation” phase, using the animal models shown in Table 1 and additional models to minimize the risk that potentially interesting compounds are missed. Throughout its history, the program has tested over 32,000 compounds from more than 600 pharmaceutical firms and other organizations and has played a major role in the development of a number of modern ASMs (Kehne et al., 2017; Porter and Kupferberg, 2017; Wilcox et al., 2020).

**FIGURE 1:**
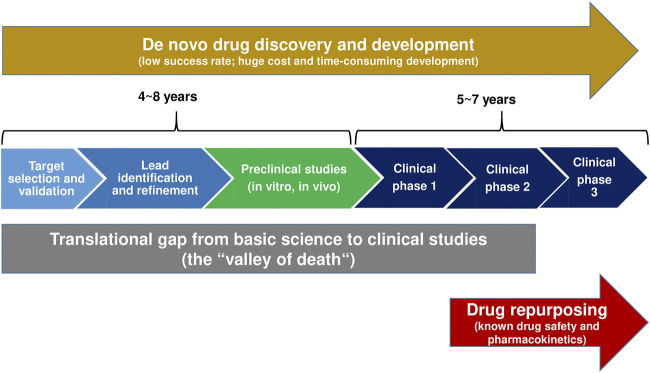
A comparison of traditional *de novo* drug discovery and development versus drug repurposing. The translation gap (“valley of death”) describes the problem of translation of basic scientific findings in a laboratory setting into human applications and potential treatments (Butler, 2008; Gamo et al., 2017). Drug repurposing reduces this gap and thus the time, risk, and investment associated with the development of new therapies.

Following the discovery and preclinical characterization of a novel ASM and clinical phase I trial for evaluation of the drug’s tolerability and pharmacokinetics, randomized, double-blind, placebo-controlled adjunctive-therapy trials in patients with drug-resistant focal seizures continue to be the primary tool to obtain regulatory approval of novel ASMs (Perucca, 2019; Boada et al., 2020). However, because the ASM market is crowded and costs of drug development are constantly increasing, the interest of industry has increased in developing novel molecules for orphan indications (i.e., rare genetic epilepsies) where unmet needs are particularly large (Perucca, 2019). In fact, five of the 11 ASMs introduced after 2005 (versus none of the 10 ASMs licensed between 1989 and 2005) have been licensed exclusively for the treatment of orphan drug indications, such as Dravet syndrome (stiripentol, cannabidiol, fenfluramine), Lennox-Gastaut syndrome (rufinamide, cannabidiol) and TSC (everolimus, cannabidiol) (Löscher and Klein, 2021a). Furthermore, as illustrated in Figure 1, repurposing (or repositioning) drugs for orphan indications by identifying new targets for FDA-approved drugs has become a successful strategy and business model in many therapeutic indications, including rare epilepsy syndromes (see *Single-Target Rather Than Multi-Target Drugs for Genetic Epilepsies: The Development of Precision Medicines*). Compared to NCEs, repurposed drugs are generally approved sooner (by 3–12 years), at reduced cost (50–60% less), and at lower risk. Indeed, approval rates for repurposed drugs are close to 30% (Fetro and Scherman, 2020) versus ≤10% for NCEs (likelihood of approval from phase I) (Hay et al., 2014; Thomas et al., 2016). Lack of efficacy is the primary reason for attrition during clinical development. Furthermore, CNS drugs have lower success rates and take a longer time to develop than do other drug classes. Nevertheless, the industry continues to develop NCEs for epilepsy therapy by the traditional drug development pipeline illustrated in Figure 1 (Löscher and Klein, 2021a), facing the translational gap that results from the often poor translatability of preclinical findings to human applications. The DML padsevonil is an excellent example of this dilemma.

### Padsevonil, the First Rationally Designed Multimodal Antiseizure Medication

As described above, at least one-third of patients with epilepsy do not respond to treatment with current ASMs (Chen et al., 2018; Devinsky et al., 2018). Patients with drug-resistant epilepsy suffer from uncontrolled seizures, which may cause injuries, disability, and increased mortality. Thus, novel ASMs with higher efficacy and tolerability that may improve adherence are urgently needed. Levetiracetam is one of the most effective drugs among the >30 ASMs that are currently available. It is thought to act mainly by modulating synaptic vesicle protein 2A (SV2A) (Lynch et al., 2004; Rogawski and Löscher, 2004), which has been demonstrated to be involved in vesicle trafficking and exocytosis, processes crucial for neurotransmission (Löscher et al., 2016). However, as will be discussed later in this review, levetiracetam is not selective for SV2A but also exerts effects on other targets (Löscher, 2020).

It was shown previously that levetiracetam administered in combination with drugs that potentiate the inhibitory effect of the neurotransmitter GABA in the brain results in a synergistic increase in antiseizure efficacy in animal models (Kaminski et al., 2009). Based on this observation, a rational medicinal chemistry design program was initiated by UCB Pharma to develop a single molecular entity that could target both SV2A and GABA_A_ receptors, resulting in the discovery of padsevonil, the first rationally designed ASM candidate that acts selectively on both pre- and postsynaptic targets (Wood et al., 2020). At recombinant SV2 proteins, padsevonil’s affinity for SV2A was greater than that of levetiracetam or brivaracetam, another SV2A ligand that has been approved for epilepsy therapy (Wood et al., 2020). Unlike the latter ASMs, padsevonil also displayed a high affinity for the SV2B and SV2C isoforms and thus is the first ligand that interacts with all three SV2 isoforms. Furthermore, padsevonil’s interaction with SV2A differed from that of levetiracetam and brivaracetam; it exhibited much slower binding kinetics: dissociation t_1/2_ 30 min compared with <0.5 min for levetiracetam and brivaracetam. At recombinant GABA_A_ receptors, padsevonil displayed low to moderate affinity for the benzodiazepine binding site, and electrophysiological studies indicated partial agonist properties at this site (Wood et al., 2020). *In vivo*, at the dose range providing seizure protection in amygdala kindling models, padsevonil occupancy of SV2A proteins was high, whereas occupancy of the benzodiazepine site was low (Wood et al., 2020). Two positron emission tomography (PET) studies were conducted in humans to evaluate occupancy at the same receptor targets after therapeutically relevant doses (Muglia et al., 2020). Modeling of these PET data confirmed a pattern similar to the preclinical model with high (>90%) sustained SV2A occupancy, and 10–15% transient GABA_A_ receptor occupancy (Muglia et al., 2020).

Leclercq et al. (2020) characterized the pharmacological profile of padsevonil in rodent seizure and epilepsy models with particular emphasis on models of difficult-to-treat or drug-resistant seizures, such as the acute 6-Hz focal seizure test in mice, as well as in the amygdala kindling model of focal epilepsy (Table 1). Padsevonil displayed robust efficacy across several validated seizure and epilepsy models, including those considered to represent drug-resistant epilepsy. In amygdala kindled mice, padsevonil was the most potent ASM compared with nine other ASMs with different mechanisms of action (MOAs). In the 6-Hz mouse model of focal seizures, padsevonil provided significantly greater protection than combinations of diazepam with levetiracetam or brivaracetam, suggesting that padsevonil’s unique MOA (modulation of SV2A, SV2B, and SV2C as well as GABA_A_ receptors) confers antiseizure properties that are superior to that derived from the combination of compounds targeting SV2A (levetiracetam, brivaracetam) and the benzodiazepine site of the GABA_A_ receptor (e.g., diazepam). Importantly, the therapeutic index of padsevovil was the highest among the ASMs tested in a mouse amygdala kindling model, indicating a favorable safety profile.

Overall, padsevonil is the first rationally designed ASM candidate that inhibits seizure activity by presynaptic modulation of all three SV2 isoforms and postsynaptic enhancement of GABA-mediated inhibition. The PET studies described above allowed the projection of a quantitatively based dosing rationale for clinical trials of padsevonil (Muglia et al., 2020). In a randomized, double-blind, placebo-controlled phase IIa proof-of-concept trial in 55 adult patients with very frequent drug-resistant focal seizures, who had failed to respond to ≥4 ASMs, significant antiseizure efficacy of padsevonil was determined during add-on administration (Muglia et al., 2020). As predicted by the preclinical data, padsevonil was generally well tolerated at doses up to 400 mg b.i.d. However, in a subsequent larger randomized placebo-controlled phase IIb add-on trial, padsevonil had only a modest effect in treatment-resistant focal epilepsy and did not separate from placebo in its primary endpoints, which optimistically included ≥75% reduction in seizure frequency (French. 2020a; Werhhan et al., 2020). Thus, the concept embraced by UCB - that their next drug development program after the approval of brivaracetam had to make a dent in treatment-resistant epilepsy - failed to materialize.

In addition to the dual-mode of action, the optimism that padsevonil would make a difference was based on the remarkable potency of this drug in models of pharmacoresistant focal epilepsy, i.e., the 6-Hz model in mice, the intrahippocampal kainate model in mice, and amygdala kindling in rats and mice (Table 1). Indeed, in most acute models, padsevonil outperformed other clinically approved ASMs. However, the typical approach of ASM testing in animal models primarily focuses on drug potency, and not efficacy (Löscher, 2016). Thus, different ASMs are compared in terms of their antiseizure ED_50_s, i.e., the dose suppressing seizures in 50% of the animals, which is calculated from dose-response curves, testing one group of animals per dose. The lower the ED_50_, the more potent is the drug, and high potency is often an important argument for selecting drugs for further development. However, it is the antiseizure efficacy that ultimately determines the clinical usefulness of a new ASM and should be considered during preclinical drug testing (Löscher, 2016). One approach in this respect is the 6-Hz mouse model, in which focal seizures are induced by transcorneal stimulation with an electrical 6-Hz current. Typically, as shown in Table 1, ASMs lose their antiseizure effect in this model if current strength is increased from 22 mA (the CD_97_ [convulsant dose in 97% of mice] in this model) to 32 mA (50% above CD_97_) and 44 mA (2 x CD_97_) (Barton et al., 2001). Thus, ASMs that remain effective at 44 mA may confer higher efficacy specifically for drug resistant patient populations in particular than ASMs that lose antiseizure activity. However, in the absence of validation by ASMs with higher clinical efficacy in drug-resistant patients, this is pure speculation, and the failure of padsevonil in the phase IIb clinical trial in patients with drug-resistant focal epilepsy seems to argue against potential predictivity of findings in the 6-Hz mouse model.

In contrast to padsevonil, the new ASM cenobamate, which was recently approved for adjunctive therapy of treatment-resistant focal epilepsy by the FDA, has demonstrated in two randomized controlled trials that it has a superior ability to render treatment-resistant focal epilepsy patients seizure-free versus any of the other novel ASMs (21% of treatment-resistant patients remained free of seizures during the 12-weeks maintenance phase) (French, 2020a,b). Yet, cenobamate was discovered purely through “standard” blind screening. When put into further development, the MOA was completely unknown, but subsequently, evidence has supported a dual mechanism of GABA enhancement and preferential inhibition of the persistent sodium channel (Löscher et al., 2021). Multi-target effects have also been described for numerous other ASMs (see next section). Thus, one explanation for the failure of padsevonil is that most ASMs already act by more than one MOA, so the dual MOA drug padsevonil does not offer anything new. Furthermore, most patients with refractory epilepsy take two, three, or more ASMs with different MOAs (Kwan et al., 2011), which renders it unlikely that a novel and rationally designed multi-target ASM is more effective than already existing ASMs or their combinations unless the novel drug acts by unique mechanisms, such as presumably those of cenobamate, which, however, may not be fully understood, yet. As shown in Table 1, cenobamate was quite effective across various preclinical models of seizures or epilepsy, including models of drug-resistant focal seizures such as the 6-Hz mouse model and amygdala kindling. Interestingly, cenobamate was about 10-times more potent than padsevonil in the MES test, whereas the opposite was true for padsevonil vs cenobamate in the 6-Hz model at 44 mA (Table 1). Thus, by the preclinical profile alone, the high clinical efficacy of cenobamate and padsevonil’s failure could not have been predicted. However, in this respect it is important to note that both padsevonil and cenobamate were administered alone during preclinical evaluation, whereas the clinical studies were performed as add-on therapy with either padsevonil or cenobamate in patients with drug resistant focal seizures. Thus, infra-additive interactions with other ASMs cannot be excluded in such trials (see *Is Multi-Target Combination Therapy (“Rational Polytherapy”) of Epilepsy More Effective Than Monotherapy With Antiseizure Medications*). Furthermore, the inclusion of patients that were resistant to other SV2A modulators (brivaracetam, levetiracetam) may have contributed to the failure of padsevonil in the phase IIb clinical trial (Werhahn et al., 2020). However, overall, the reasons for the apparent dichotomy in clinical efficacy between padsevonil and cenobamate remain largely a mystery.

## Most Antiseizure Medications Already Act by More Than One Mechanism

**FIGURE 2:**
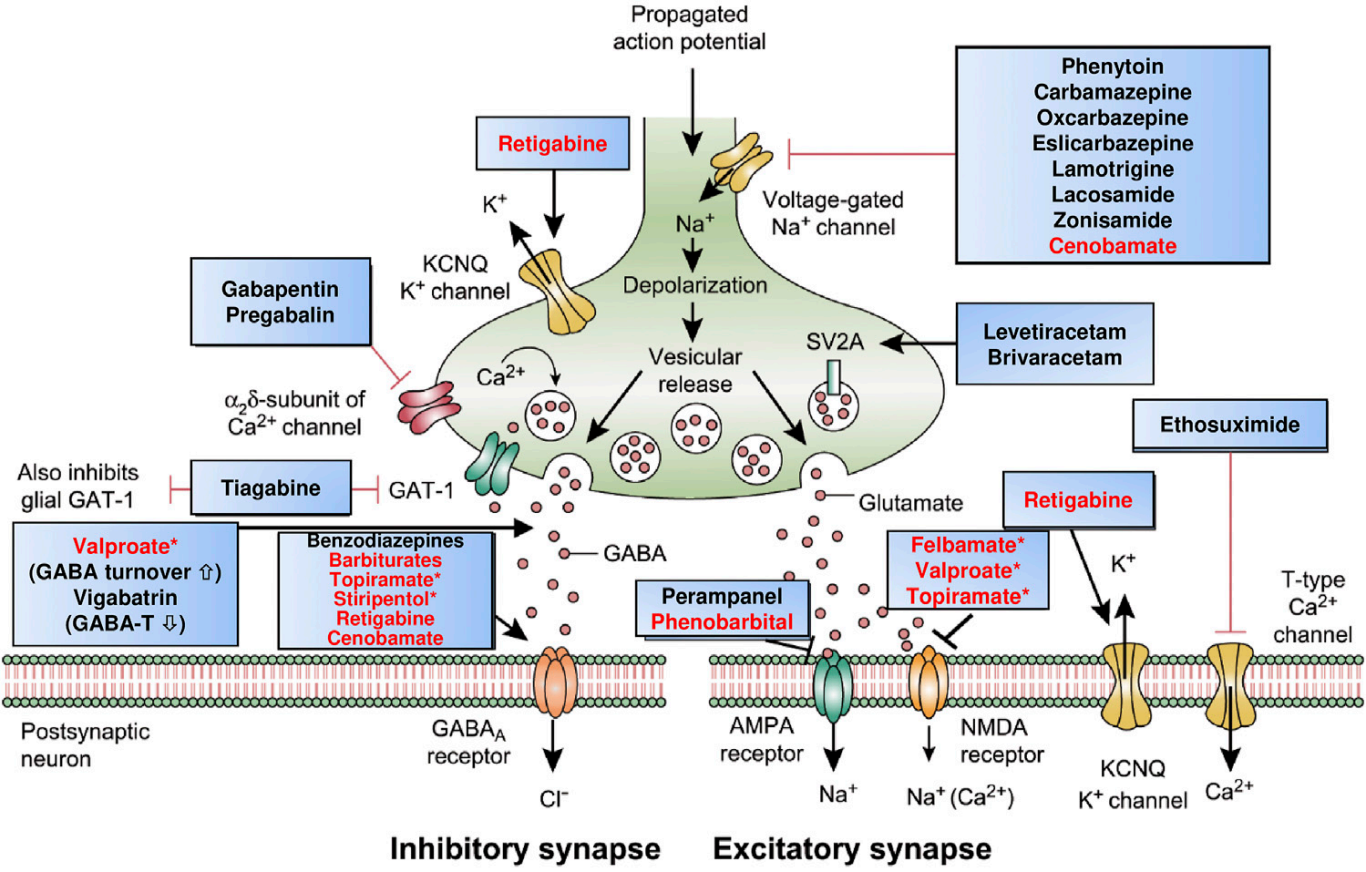
Mechanism of action of clinically approved antiseizure medications (ASMs). Updated and modified from Löscher and Schmidt (2012), Löscher et al. (2016), and Löscher and Klein (2021b). Drug names highlighted in red indicate that these compounds act by multiple mechanisms; asterisks indicate that not all mechanisms are shown here. Note that several of the ASMs presumed to act by a single mechanism (e.g., phenytoin, carbamazepine, gabapentin) also possess other mechanisms (see Table 2). Some ASMs, e.g., fenfluramine, are not shown here, but their mechanism(s) of action are described in Table 2. Abbreviations: AMPA, α-amino-3-hydroxy-5-methyl-4-isoxazolepropionic acid; GABA, γ-aminobutyric acid; GABA-T, GABA aminotransferase; GAT-1, GABA transporter 1; KCNQ, Kv7 potassium channel family; NMDA, N-methyl-d-aspartate.

There are at least three strategies that have been used for the development of ASMs: 1) random (or phenotypic) screening of newly synthesized chemical compounds of diverse structural categories for antiseizure activity, 2) structural variation of known ASMs (i.e., a chemocentric approach), and 3) rational target-based drug design (or rational drug development) based on knowledge of the perceived pathophysiological events responsible for epileptic seizures (Löscher and Schmidt, 1994; Bialer, 2002; Löscher et al., 2013; Löscher and Klein, 2021a). Furthermore, serendipity played a significant role in the development of several ASMs, including phenobarbital, valproate, topiramate, levetiracetam, and fenfluramine (Porter and Rogawski, 1992; Maryanoff, 2009). All these strategies have generated clinically useful ASMs, although many scientists believe that the strategy of rational (‘modern’) drug development has important advantages over the more traditional other strategies (Porter and Rogawski, 1992; Löscher and Schmidt, 1994; Meldrum and Rogawski, 2007; Palestro et al., 2018; Gantner et al., 2021). Examples of rationally developed single-targets drugs are the GABA uptake inhibitor tiagabine, the SV2A modulator brivaracetam, the AMPA receptor antagonist perampanel, and the GABA degradation inhibitor vigabatrin (Tables 1 and 2; Figure 2). However, the majority of the about 30 clinically approved ASMs were not developed by rational target-based strategies, so the MOA was only discovered after the drugs were developed based on their antiseizure efficacy in available preclinical models.

The actions of most ASMs on molecular targets can be categorized into four broad groups (Rogawski et al., 2016; Sills and Rogawski, 2020): 1) modulation of voltage-gated ion channels, including sodium, calcium, and potassium channels; 2) enhancement of GABA-mediated inhibition through effects on GABA_A_ receptors, the GAT1 GABA transporter, GABA transaminase, or the GABA synthesizing enzyme glutamate decarboxylase (GAD); 3) inhibition of synaptic excitation mediated by ionotropic glutamate receptors, including N-methyl-d-aspartate (NMDA) and α-amino-3-hydroxy-5-methyl-4-isoxazole-propionate (AMPA) receptors; and 4) direct modulation of synaptic release through effects on components of the release machinery, including SV2A and the α2δ subunit of voltage-gated calcium channels (Figure 2; Table 2). The result of the interactions at these diverse targets is to modify the intrinsic excitability properties of neurons or to alter fast inhibitory or excitatory neurotransmission. By these actions, ASMs reduce the probability of seizure occurrence by modifying the bursting properties of neurons (reducing the capacity of neurons to fire action potentials at a high rate) and reducing synchronization in localized neuronal ensembles. In addition, ASMs inhibit the spread of abnormal firing to adjacent and distant brain sites.

As shown in Figure 2 and Table 2, most ASMs act by more than one MOA and thus can be considered multi-target drugs. Furthermore, even many of those ASMs that are typically presented as single-target drugs (Table 1) presumably act by more than one MOA (Rogawski et al., 2016; Sills and Rogawski, 2020). As shown in Table 2, examples are the sodium channel modulator phenytoin, which also blocks high-voltage activated Ca^2+^ channels, or the Kv7 (KCNQ) potassium channel opener retigabine, which also acts as an allosteric positive modulator (PAM) at GABA_A_ receptors. The latter drug is a good example of how more modern technology improves our understanding of the MOAs of ASMs (Löscher et al., 2021). Thus, retigabine was long thought to act exclusively via activation of Kv7 potassium channels (Gunthorpe et al., 2012), but recent evidence that inhibitory effects of retigabine on seizure-like activity in hippocampal neurons persist in the presence of a blockade of Kv7 channels has bolstered the view that positive modulation of GABA_A_ receptors likely makes a significant contribution to its antiseizure activity (Treven et al., 2015). This view is reinforced by the observation that, at lower concentrations than those required for effects on synaptic GABA_A_ receptors, retigabine selectively enhances GABA-mediated inhibition by extrasynaptic GABA_A_ receptors that contain the δ-subunit (Treven et al., 2015).

**FIGURE 3:**
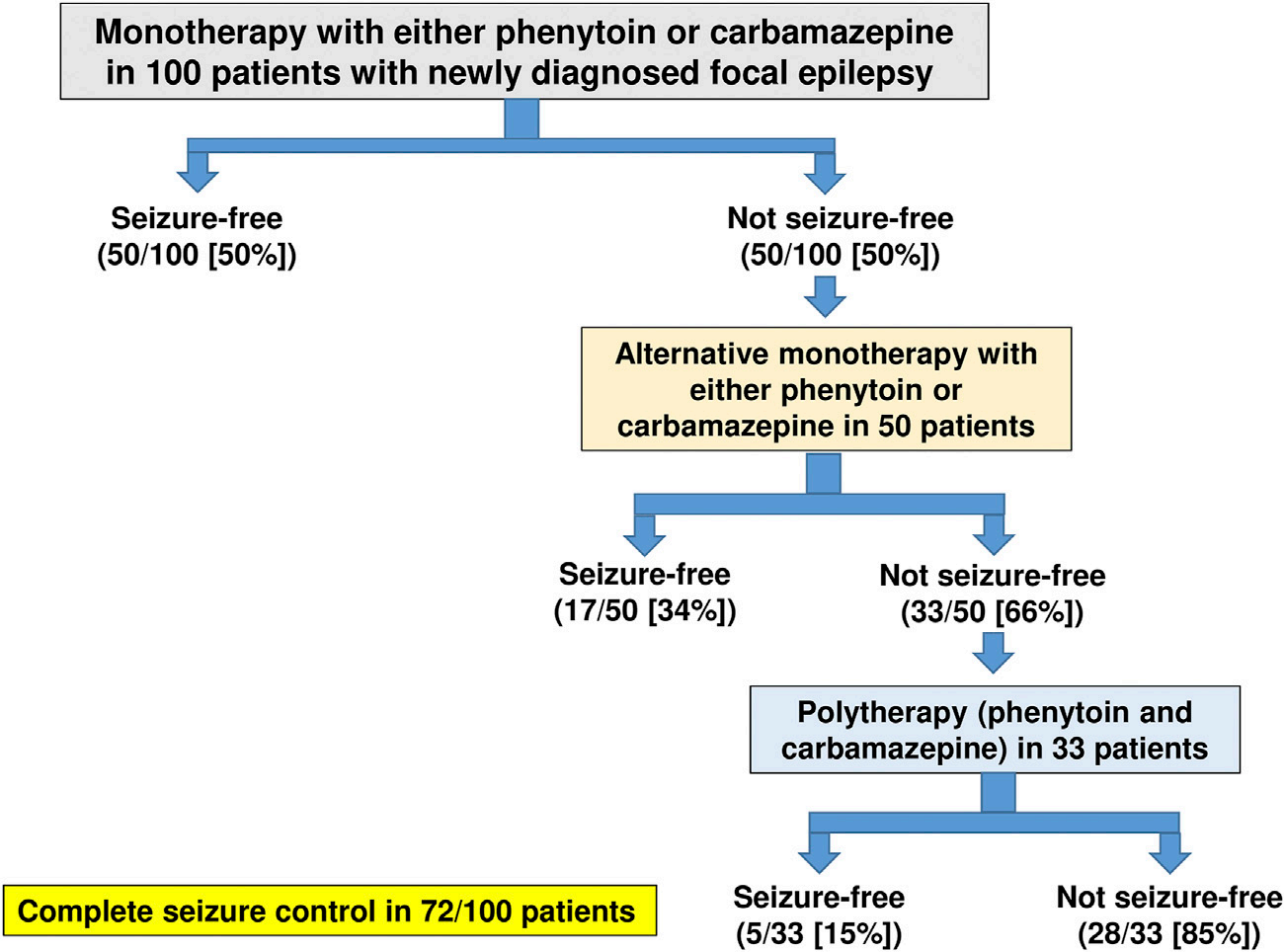
Summary of the efficacy of monotherapy (with phenytoin or carbamazepine), alternative monotherapy (with the alternative agent), and polytherapy (with both agents) in 100 adult patients with newly diagnosed focal epilepsy. Success was defined as complete seizure control. Data are from a randomized, nonblinded trial in adult patients with newly diagnosed partial epilepsy (Hakkarainen, 1980).

An excellent example of how the allocation of ASMs to perceived or simplified mechanistic categories can lead to false conclusions is illustrated in Figure 3. In a randomized, nonblinded trial in 100 adult patients with newly diagnosed focal epilepsy published only in abstract form (Hakkarainen, 1980), 50 patients were randomized to receive phenytoin monotherapy and 50 to carbamazepine monotherapy. As Figure 3 shows, the first treatment with monotherapy at maximally tolerated doses resulted in complete seizure control in 50% of the 100 patients, while treatment failed in 50 patients. These latter patients were transferred to the alternative drug given alone. If alternative (or sequential) monotherapy failed (as it did in 33 patients), both drugs were given together. This trial showed convincingly that subsequent sequential monotherapy with the alternative agent achieved complete seizure control in 17 of 50 patients (34%) of patients in whom previous monotherapy had failed (Figure 3). In addition, it was demonstrated that patients who had failed to respond to alternative monotherapy responded to polytherapy at a rate of 5 of 33 patients (15%). If both phenytoin and carbamazepine acted exclusively by the same MOA, i.e., modulation of voltage-dependent sodium channels, one would not have expected that subsequent monotherapy with either alternative drug would have been effective in patients that were resistant to the prior drug; instead the opposite was the case. Similar findings of the efficacy of alternative monotherapy were reported for carbamazepine and vigabatrin (Schmidt and Gram, 1995). Yet, the perceived MOAs of these agents are quite different (sodium channel blockade versus GABA metabolism; Figure 2 and Table 2), so it is not surprising that sequential treatment with two ASMs that act by different mechanisms would lead to increased seizure freedom. This finding has been substantiated by several large clinical studies (Mattson et al., 1986; Beghi et al., 2003; Millul et al., 2013; Semah et al., 2014).

## Is Multi-Target Combination Therapy (“Rational Polytherapy”) of Epilepsy More Effective Than Monotherapy With Antiseizure Medications?

An alternative to multi-target drugs or DMLs in the treatment of epilepsy would be to combine ASMs with different MOAs, which has been termed “rational polypharmacy” or “rational polytherapy” (Ferrendelli, 1995; Leppik, 1996). Indeed, most patients with refractory epilepsy take two, three, four, or even more ASMs (Kwan et al., 2011), however, these combinations are not always chosen on rational grounds. There are different possible approaches to polypharmacy (Morphy and Rankovic, 2005). Traditionally, clinicians have treated unresponsive patients by combining therapeutic mechanisms with cocktails of drugs. Most frequently, the cocktail is administered in the form of two or more individual tablets. However, the benefits of this approach are often compromised by poor patient compliance. In the last ∼20 years, there have been attempted moves toward multicomponent drugs whereby two or more agents are co-formulated in a single tablet in a fixed-dose combination to make dosing regimens simpler, thereby improving patient compliance (Morphy and Rankovic, 2005; Wertheimer, 2013; Esba et al., 2021). Several such multicomponent drugs have been launched for diverse indications, but clinicians might still prefer prescribing combinations of existing monotherapies that may offer greater dose flexibility and lower cost treatment, particularly in the case of generic drugs. In contrast to many other diseases, fixed-dose multicomponent drugs do not play any role in epilepsy therapy. An alternative strategy (discussed above) is to develop a single chemical entity, i.e., a multimodal drug or DML that can modulate multiple targets simultaneously. However, the example of padsevonil illustrates that the latter approach is not necessarily more effective than more conventional approaches of polypharmacy.

**FIGURE 4:**
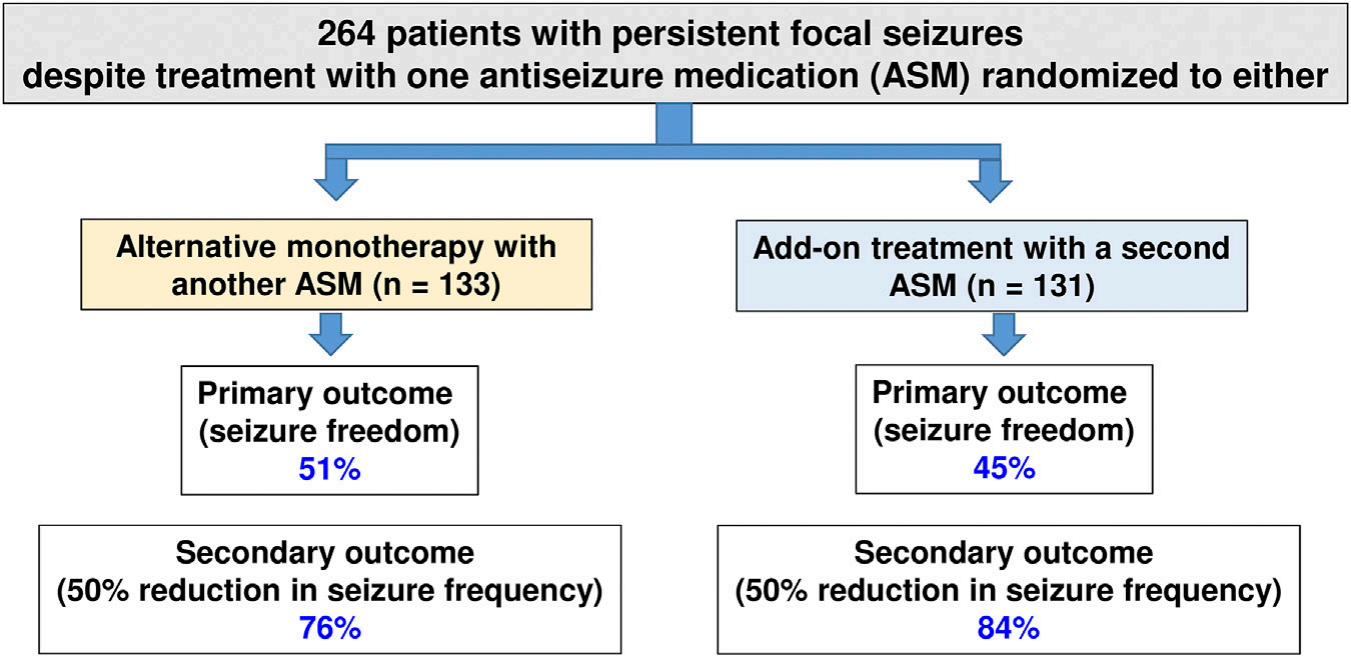
Efficacy of early add-on treatment versus alternative monotherapy in patients with persistent focal seizures despite treatment with one antiseizure medication (ASM), which was the first ASM administered in these patients. Alternative monotherapy or early initiation of combination therapy with another ASM resulted in similar efficacy, and the adverse effects associated with monotherapy or polytherapy were similar. Data are from a multicentre, cluster-randomized, prospective, controlled trial in patients with persistent partial seizures despite treatment with one ASM (which was the first ASM in these patients). For details, see Semah et al. (2014).

The principle underlying rational polypharmacy is that the combination of two medications with differing mechanisms of action may result in supra-additive or synergistic antiseizure effects, with infra-additive toxicity (Ferrendelli, 1995; Leppik, 1996). Although there is increasing evidence that this may be a reasonable approach to managing drug-resistant epilepsy (Brodie, 2016), only a few studies compared the efficacy of rational polypharmacy with the efficacy of alternative (or sequential) monotherapy at maximally tolerated doses. One example of such a study is illustrated in Figure 4, demonstrating that in patients with persistent focal seizures despite treatment with one ASM, administration of alternative monotherapy is as effective as an add-on treatment with a second ASM (Semah et al., 2014).

**FIGURE 5:**
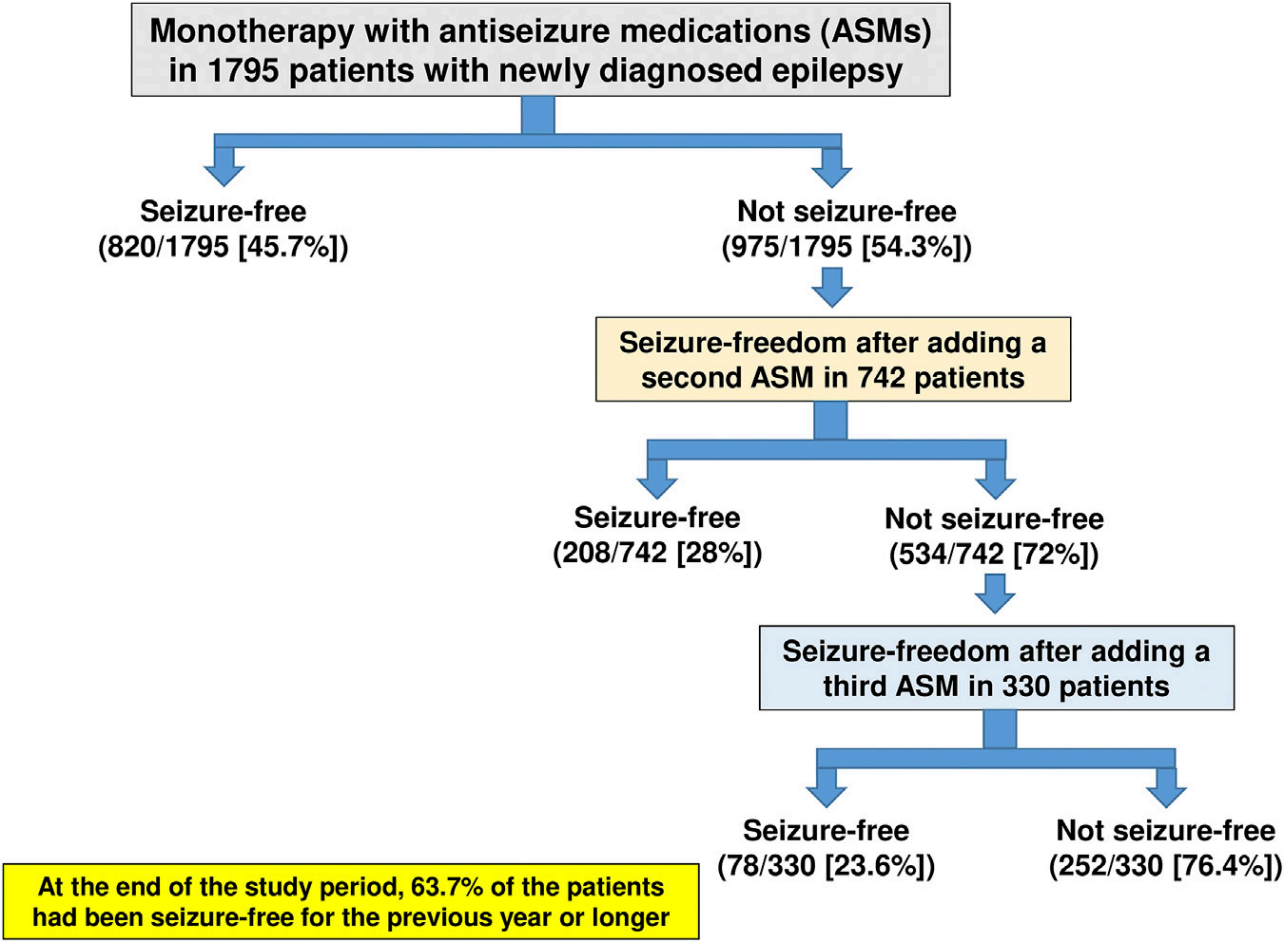
Long-term treatment outcome in patients with newly diagnosed and treated epilepsy. Data are from a longitudinal observational cohort study conducted at the Epilepsy Unit of the Western Infirmary in Glasgow, Scotland, over a period of 30 years. For details, see Chen et al. (2018).

In the largest clinical study of its kind, a longitudinal observational cohort study conducted at the Epilepsy Unit of the Western Infirmary in Glasgow, Scotland, a total of 1795 individuals were newly treated for epilepsy with ASMs between July 1, 1982, and October 31, 2012 (Chen et al., 2018). As shown in Figure 5, 45.7% of these patients became seizure-free on their first ASM regimen. If this first ASM failed, the second and third regimens provided an additional 11.6 and 4.4% likelihoods of seizure freedom, respectively, demonstrating the relatively low impact of drug combination therapy. At the end of the study period, 1,144 patients (63.7%) had been seizure-free for the previous year or longer (Chen et al., 2018). Thus, most patients who attained seizure control did so with the first or second ASM. Furthermore, despite the increased use of many new ASMs with differing MOAs over the past 2 decades, long-term outcomes in adolescent and adult patients who are diagnosed with common, newly diagnosed epilepsies have not improved, but drug resistance is still in the range of 30–40% (Löscher and Schmidt, 2011; Chen et al., 2018) although cenobamate may be changing this.

Given this reality, there has been renewed interest in understanding and designing effective ASM regimens utilizing two (or more) medications, i.e., rational polypharmacy (Brodie and Sills, 2011; Brigo et al., 2013; Barker-Haliski et al., 2014; Gidal, 2015; Verrotti et al., 2020a, Verrotti et al., 2020b). Perhaps just as important, this concept also has the potential to inform the clinician of “irrational” polypharmacy, in other words, combinations of ASMs that yield antagonistic effects on seizures, or perhaps supra-additive adverse effects (Gidal, 2015). The inherent problem with the concept of rational (or irrational) polypharmacy is that the number of clinically approved ASMs is so high that several hundred dual therapies and more than 1,000 triple combinations are possible, making any systematic evidence-based clinical evaluation impossible. This issue becomes even more complex when considering the multiple possible dose ratios between two or more ASMs in combination. Therefore, a systematic evaluation of ASM combinations is only feasible in animal models (Deckers et al., 2000; Verrotti et al., 2020a). Preclinical combination studies in seizure models should incorporate efficacy and toxicity readouts, in which both drugs are at least minimally effective, use drug ratios that reflect those employed clinically, include drug concentration analysis in both plasma and brain to rule out confounding pharmacokinetic interactions, and employ an appropriate method of analysis such as isobolography (Brodie and Sills, 2011). Isobolography is preferable as it provides a robust measure of effectiveness and affords a definitive determination of infra-additive (antagonistic), additive, or supra-additive (synergistic) interactions. The ideal ASM combination displays pharmacological synergism, whether it is defined as improved efficacy with similar toxicity, similar efficacy with reduced toxicity, or, ideally, improved efficacy with reduced toxicity.

By using such criteria, hundreds of different ASM combinations have been evaluated preclinically (Deckers et al., 2000; Czuczwar et al., 2009; Blaszczyk et al., 2018; Verrotti et al., 2020a). Table 3 summarizes those duotherapies that exerted synergistic (supra-additive) antiseizure efficacy in animal models and/or presumed synergistic efficacy in clinical trials. To our knowledge, the first preclinical study on systematically tested ASM combinations was published by Weaver et al. (1955), showing synergistic antiseizure efficacy of phenytoin and phenobarbital combinations in the MES test in rats. Clinical studies seemed to substantiate the beneficial interaction of this combination (Table 3). Furthermore, available clinical studies on several other ASM combinations are quite consistent with the data generated from animal studies (Table 3). However, most of the clinical data summarized in Table 3 are from small studies, often not replicated independently, and there are almost no randomized controlled studies comparing different ASM combinations (Verrotti et al., 2020b). The best nonrandomized, controlled data in favor of true synergism exist for valproate and lamotrigine for focal-onset and generalized seizures (Kwan and Brodie, 2006). Brodie and Yuen (1997), who performed a large, 347-patient study designed to assess the efficacy of lamotrigine monotherapy, evaluated an interim study epoch during which both lamotrigine and the baseline drug, which was carbamazepine, phenytoin, or valproate, were used in combination. Mean seizure reductions at the end of this epoch were: lamotrigine–valproate 83%, lamotrigine–carbamazepine 43%, and lamotrigine–phenytoin 34%, thus demonstrating the superiority of the lamotrigine-valproate combination. In line with this finding, Poolos et al. (2012) retrospectively examined treatment records of adults with highly refractory epilepsy to determine whether any combinations of eight of the most commonly used ASMs possessed superior efficacy and found that, out of the 32 most frequently used ASM combinations, only the combination of lamotrigine and valproate had superior efficacy.

**TABLE 3:** Experimental and clinical evidence for synergistic (supra-additive) two-drug combinations of antiseizure medications. For references see Deckers et al. (2000), Kaminski et al. (2009), Blaszczyk et al. (2018), and Verrotti et al. (2020a,b). “?” indicates that no data were found. Only few data are available on the combinations of more than two antiseizure medications (not shown here).

Drug combination	Synergistic antiseizure effect in animal models	Presumed synergistic antiseizure effect in patients with epilepsy^a^	Pharmacokinetic interactions
Carbamazepine + lacosamide	+	?	No
Carbamazepine + phenytoin	?	+	Yes
Carbamazepine + valproate	No (only additive)	+	Yes
Carbamazepine + vigabatrin	?	+	Minor
Gabapentin + tiagabine	+	?	No
Gabapentin + lacosamide	+	?	No
Gabapentin + carbamazepine	+	?	Yes
Gabapentin + oxcarbazepine	+	?	Yes
Gabapentin + phenytoin	+	?	Yes
Gabapentin + valproate	+	?	No
**Lamotrigine + valproate**	**+**	**+**	**No**
Lamotrigine + phenobarbital	+	+	Yes
Lamotrigine + lacosamide	+	+	No
Levetiracetam + lacosamide	+	+	No
Levetiracetam + valproate	+	?	No
Levetiracetam + clonazepam	+	?	No
Levetiracetam + phenobarbital	+	?	?
Levetiracetam + topiramate	+	?	No
Phenytoin + phenobarbital	+	+	Yes
Stiripentol + carbamazepine	+	?	Yes
Topiramate + felbamate	+	?	No
Topiramate + gabapentin	+	?	No
Topiramate + lamotrigine	+	+	No
Topiramate + oxcarbazepine	+	?	No
Topiramate + lacosamide	+	No (only additive)	No
Valproate + phenytoin	+	?	Yes
Valproate + ethosuximide	No (only additive)	+	Yes

a Note that the combination of lamotrigine with valproate is the only synergistic combination with sufficient clinical evidence (see text).

Margolis et al. (2014) proposed that in adult epilepsy patients, persistence on therapy could be a valid surrogate marker of overall treatment effectiveness (efficacy + tolerability). In this retrospective evaluation in over 8,000 patients with focal-onset epilepsy, ASMs were classed as sodium channel blockers, GABAergic drugs, SV2A modulators, or mixed mechanisms. In those patients receiving combinations solely of GABAergic drugs, or solely of sodium channel blockers, persistence on therapy was the shortest as compared with those receiving combinations of ASMs with differing primary mechanisms. In particular, combinations including levetiracetam (SV2A) demonstrated significantly longer persistence as compared with single-mechanism combinations (Margolis et al., 2014). Despite the limitations of this type of approach, this analysis was quite consistent with data generated from animal studies (Table 3). However, as pointed out by Gidal (2015), this study did not provide definitive proof of rational polytherapy.

Overall, polypharmacy is clinically useful in a minority of subjects with drug-resistant epilepsy (Chen et al., 2018), but despite being a standard treatment strategy for over one hundred years, it has been poorly studied clinically (Schmidt, 2016). In fact, there are no evidence-based data that show a significant difference in seizure control between monotherapy and polytherapy (Schmidt, 2016). For instance, in a multicenter double-blind randomized clinical trial in 130 adult patients with untreated generalized tonic-clonic and/or focal seizures, in which patients were randomized to carbamazepine monotherapy or carbamazepine plus valproate polytherapy, no statistical differences were found between the two treatments in the reduction of seizure frequencies, in overall neurotoxicity, or overall systemic toxicity (Deckers et al., 2001). Thus, it appears that despite decades of research, monotherapy is still preferable to polypharmacy, rational or not, in most patients with epilepsy. As discussed above, one explanation for this conclusion is that most ASMs already act by several MOAs. However, there has also been a debate on i) whether what we know about MOAs of ASMs matches what we know about seizure generation; ii) whether MOA predicts ASM efficacy; iii) whether MOA matters when alternative monotherapy or combinations of ASMs are used; iv) whether ASMs with novel MOAs are more effective than drugs acting by established mechanisms; and v) whether addition of an ASM with a new MOA to ASMs with established MOAs has added therapeutic benefit (Brodie et al., 2011; Perucca, 2011; Schmidt 2011). Intuitively, the answer to these questions would be “yes”, but in reality, these are quite complex issues that go beyond the scope of the present review.

## Single-Target Rather Than Multi-Target Drugs for Genetic Epilepsies: The Development of Precision Medicines

**FIGURE 6:**
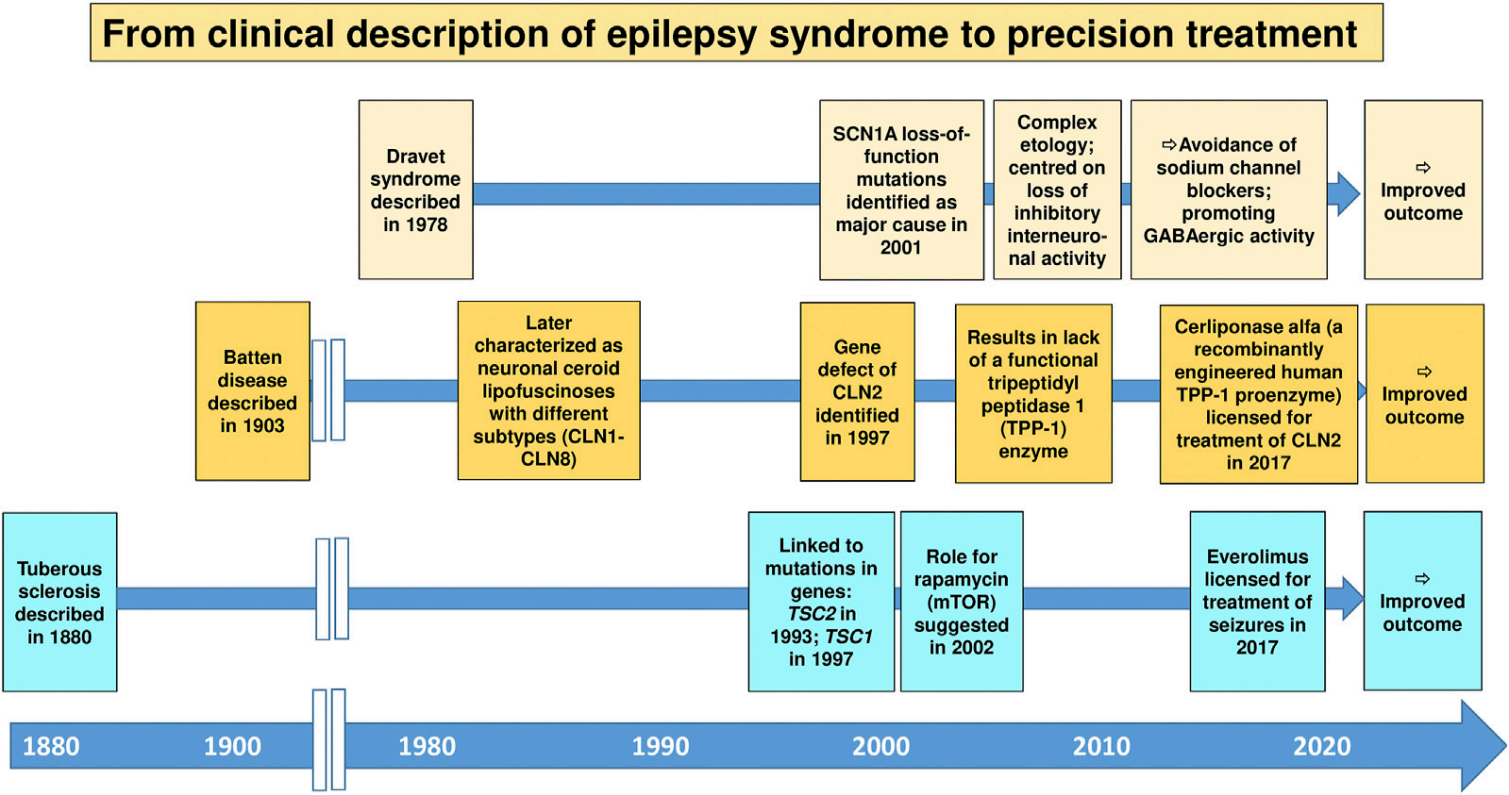
The historical development of precision medicine, i.e., the treatment of patients with therapy targeted to their specific pathophysiology, for genetic epilepsies. Three important examples are shown. For details, see Sisodiya (2021) and Specchio et al. (2020).

In the context of epilepsy therapy, precision medicine may be defined as the treatment of patients with therapy targeted to their specific pathophysiology (Demarest and Brooks-Kayal, 2018). Furthermore, individual variability in genes, environment, and lifestyle may be taken into account in precision medicine (National Research Council (US) Committee on A Framework for Developing a New Taxonomy of Disease, 2011). The idea of precision medicine or the closely related concept of personalized medicine is not new, but the introduction of pharmacogenomics/pharmacogenetics into clinical care has added an entirely new dimension to the term “personalized medicine” (Waldman and Terzic, 2008; Dickmann and Ware, 2016). Precision medicine is currently a keen area of basic and medical research across both academia and industry (Dickmann and Ware, 2016). In the epilepsies, precision medicine has gathered much attention, especially with gene discovery pushing forward a new understanding of disease biology (Demarest and Brooks-Kayal, 2018; Löscher et al., 2020; Sisodiya, 2021). Enthusiasm for precision medicine currently stems largely from discoveries from genetics about the causation of some of the rare, severe, typically early-onset epilepsies, including the developmental and epileptic encephalopathies (Sisodiya, 2021). These discoveries have in some cases led to a better understanding of disease biology, and, occasionally, rational treatment strategies have been devised, including a better selection from existing ASMs or repurposing of drugs previously not approved for use in epilepsy, sometimes with dramatic responses (Demarest and Brooks-Kayal, 2018; Moller et al., 2019; Löscher et al., 2020). The recent approval of fenfluramine for the treatment of Dravet syndrome is an important example (Gogou and Cross, 2021), although one may argue that the specific MOA of this drug is not sufficiently understood. However, this enthusiasm needs to be tempered by the fact that most reports on beneficial effects of repurposed drugs are anecdotal and short-term, and that for many of the newly-explained genetic epilepsies, a precision medicine approach employing a theoretically ideal treatment is not available, or in fact fails (Sisodiya, 2020; Sisodiya, 2021). Nevertheless, the approach of identifying the cause of a particular epilepsy and establishing a rational treatment option remains attractive and may offer a novel strategy for epilepsies that were previously resistant to treatment. Perhaps the best examples of this are TSC, Dravet syndrome, and neuronal ceroid lipofuscinosis type 2 (CLN2) (Figure 6).

TSC is a rare genetic neurocutaneous disorder with epileptic seizures as a common and early presenting symptom. The rationale for using of everolimus, an inhibitor of the mammalian target of rapamycin (mTOR), for treatment of seizures in patients with mutations in TSC genes was based on findings that these mutations are causally linked to activation of the mTOR signaling cascade (Jeong and Wong, 2016). However, as shown in Figure 6, it took almost 140 years from the clinical description of the TSC syndrome in humans to approval of everolimus as a precision medicine for this devastating disease. This was different with Dravet syndrome, a rare, drug-resistant epilepsy that begins in the first year of life in an otherwise healthy infant. For this syndrome, the period from the clinical description to mechanism-based (“precision”) therapy was less than 30 years (Figure 6). Moreover, the broad clinical utility of everolimus and other rapalogues for the treatment of a diversity of epilepsy syndromes remains to be further determined.

Neuronal ceroid lipofuscinoses (Batten disease) are a group of inherited disorders caused by deficiencies in lysosomal enzymes in which there is progressive intellectual and motor function deterioration with refractory seizures (Junaid and Pullarkat, 2001). For many decades, no effective treatment was available for this group of devastating neurodegenerative disorders. One of these conditions, CLN2, is caused by the lack of a functional tripeptidyl peptidase 1 (TPP-1) enzyme, which serves as a lysosomal exopeptidase that acts on a broad range of protein substrates. Based on the pathophysiology of CLN2, recombinant human tripeptidyl peptidase 1 (cerliponase alfa) has been developed as an enzyme replacement to CLN2 (Schulz et al., 2018). Cerliponase alfa treatment has been demonstrated to slow the progressive motor deterioration in CLN2 disease and improve survival (Schulz et al., 2018). This precision medicine treatment was licensed in 2017, i.e., ∼110 years after the clinical description of Batten disease (Figure 6).

Several other examples of precision (mechanism-based) treatments have been discussed recently (Löscher et al., 2020; Sisodiya, 2021), although the clinical evidence is often limited, yet. As a result of the recent advances in the ‘omics’, deep-phenotyping techniques, and genetic epilepsy models, the path from disease description to gene discovery to first treatment with an etiology-based precision medicine is now much shorter than in the examples illustrated in Figure 6. As illustrated by the example of everolimus for the treatment of TSC or cerliponase alfa for CLN2, precision medicine is typically single-target rather than multi-target, although multi-target approaches may be needed for genetic epilepsies with complex molecular mechanisms. The example of everolimus for TSC therapy also illustrates that precision medicine represents more than just symptomatic suppression of seizures, as is the case of currently available ASMs. Indeed, it has been suggested that everolimus does not only suppress seizures in patients with TSC but may also have the potential to be a disease-modifying therapy in this disease (Jeong and Wong, 2016). Everolimus has demonstrated significant reductions in tumor volume in subependymal giant cell astrocytomas (SEGAs) associated with TSC, which is a disease-modifying effect. However, there is no evidence yet of a positive effect of everolimus on the cognitive and neuropsychiatric deficits in TSC patients or of a lasting disease modification of epilepsy that persists after withdrawal of the treatment (Overwater et al., 2019). Finally, it is important to note that precision medicine approaches are not always available, and not always successful (Sisodiya, 2021). However, molecular medicine is an important path forward from the current curative model of patient care to preventive medicine in patients at risk.

## Single-Target Drugs Versus Multi-Target Drugs Versus Multi-Target Drug Combinations for the Prevention of Epilepsy in Patients at Risk

**FIGURE 7:**
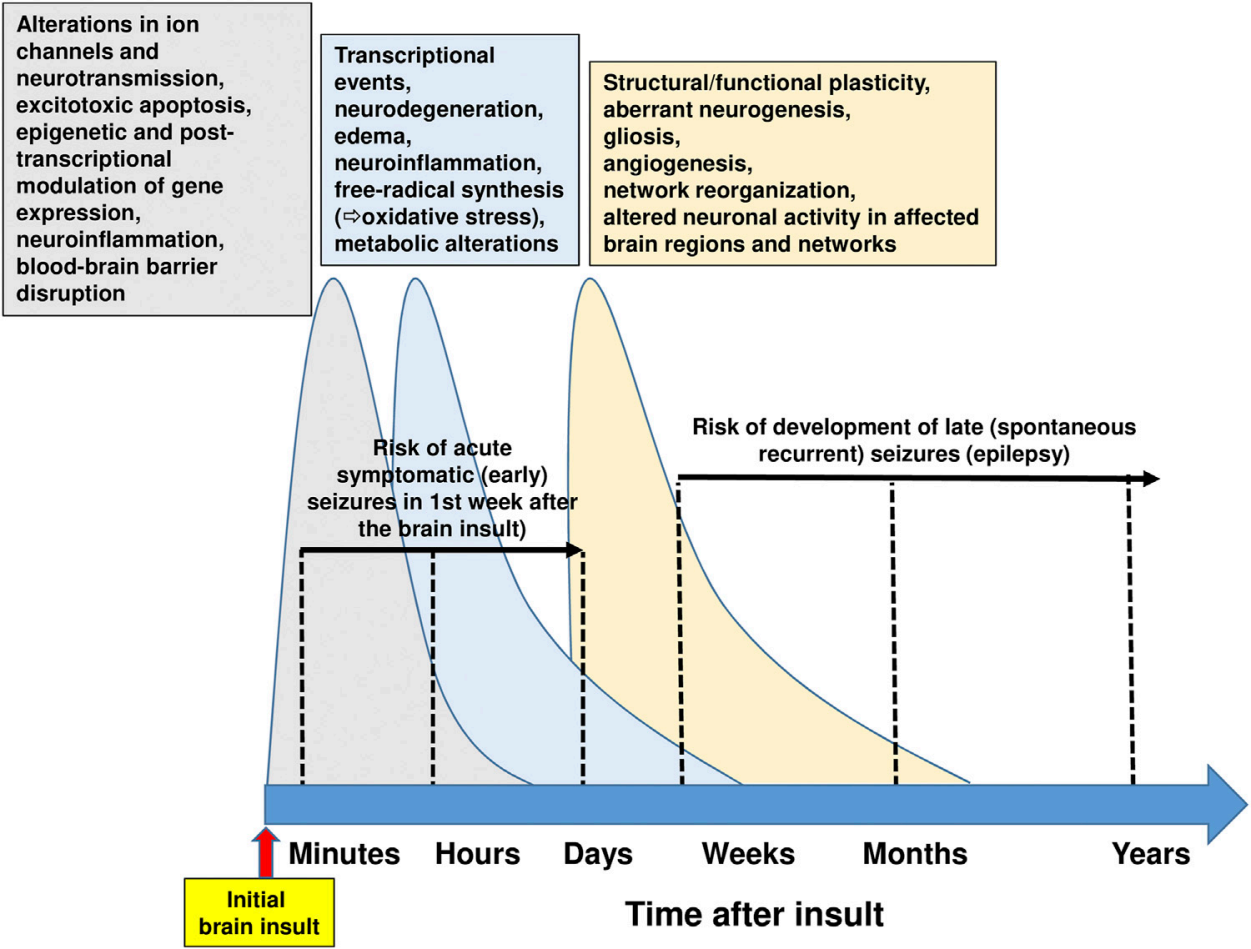
Time course of pathophysiological brain alterations after an initial brain insult. Initial brain injuries comprise traumatic brain injury, stroke, hypoxic-ischemic encephalopathy, brain infections, or prolonged seizures such as complex febrile seizures or status epilepticus. The complex molecular, structural and functional alterations, which underlie the motor, cognitive, and behavioral abnormalities and the spontaneous recurrent seizures (i.e., epilepsy) that may develop after brain injury, exhibit commonalities across different brain insults, thus providing an opportunity for therapeutic intervention. Following brain injuries as illustrated here, the cascade of events that are primarily suggested by experimental evidence can be classified temporally following the initial insult into 1) early changes (including post-translational modification of receptor and ion-channel related proteins), which occur within seconds to minutes; 2) more retarded changes (e.g., neuronal death, inflammation, altered transcriptional regulation of genes), which occur within hours to days; and 3) later changes (including morphological alterations such as mossy fiber sprouting, gliosis, and neurogenesis), occurring days to weeks to months after the initial insult. These molecular, structural and functional alterations eventually lead to structural and functional changes in neurons and neuronal networks, which are eventually manifested as abnormal hyperexcitability and spontaneous seizures. Note that acute symptomatic (early) seizures that may occur in the first week after brain injury are not considered spontaneous seizures. Current drug therapy of spontaneous seizures is symptomatic in that available antiseizure medications inhibit seizures, but neither effective prophylaxis nor cure is available. The complexity of the alterations illustrated in this figure indicates that multi-targeted drug combination therapy is needed for disease modification. However, please note that part of the multiple changes after brain injury may be aimed to repair or reverse the detrimental consequences of the insult. For instance, secondary neuroinflammation both promotes further injury, resulting in cell death, but conversely plays a beneficial role, by promoting recovery (Jayakar et al., 2019). For details see Rakhade and Jensen (2009), Clossen and Reddy (2017), Klein et al., 2018), Somayaji et al. (2018), Campbell et al., 2019, and Löscher (2020).

Treatment of epilepsy with ASMs is purely symptomatic, i.e., does not alter the natural history of epilepsy or its progression (Devinsky et al., 2018). Thus, drugs or drug combinations that exert disease-modifying activity or prevent epilepsy in patients at risk are urgently needed (Löscher et al., 2013; Klein and Tyrlikova, 2020; Löscher, 2020). At least 20% of all epilepsies are caused by acute CNS insults (Banerjee et al., 2009). In the U.S., traumatic brain injury (TBI) causes approximately 6% of all epilepsies, cerebrovascular accident (CVA) 11%, infections 4%, and new-onset cryptogenic status epilepticus (SE) < 1% (Hauser et al., 1993). The ability to prevent epilepsy after brain injury or reduce its severity is one of the great unmet needs in neurology (Pitkänen and Lukasiuk, 2011; Löscher et al., 2013; Klein and Tyrlikova, 2020). A latent period of months to years often exists between the acute insult and the onset of clinically obvious epilepsy, thus offering a window of opportunity to interfere with the process (termed epileptogenesis) leading to epilepsy (Pitkänen et al., 2015). Similarly, epilepsy may develop after a variety of gene mutations that are often known before the onset of epilepsy, thus providing an opportunity to prevent or modify epilepsy by early treatment (Perucca and Perucca, 2019). Several treatments aimed at correcting specific pathogenic defects responsible for rare genetic epilepsies are currently in development, and range from traditional small molecules to novel approaches involving peptides, antisense oligonucleotides, and gene therapy (Perucca and Perucca, 2019). However, in patients developing epilepsy after brain injury, epileptogenesis is a complex multifactorial process, involving inflammation, neuron loss, plasticity, and circuit reorganization (Figure 7) (Klein et al., 2018). Thus, it is unlikely that a drug that selectively interferes with only one specific target in the epileptogenic process will prevent epilepsy or modify its course in patients at risk (Löscher, 2020).

A variety of compounds, including several ASMs, have been evaluated for antiepileptogenic effects in animal models of acquired epilepsy (Löscher and Brandt, 2010; Pitkänen and Lukasiuk, 2011; Löscher, 2020). Furthermore, a series of clinical posttraumatic epilepsy (PTE) prevention trials have been performed, administering drugs such as phenytoin, carbamazepine, phenobarbital, valproate, or the NMDA receptor antagonist magnesium sulfate during the latent period (Temkin, 2001; Temkin, 2009; Trinka and Brigo, 2014; Thompson et al., 2015). All these trials have been negative, which, at least in part, is consistent with preclinical data in models of acquired epilepsy (Table 4). In only a few clinical trials, drug combinations (mainly phenytoin and phenobarbital) were evaluated, but none of these studies have shown reliable evidence that they prevent epilepsy after TBI (Temkin, 2001; Temkin, 2009). As shown in Table 4, there is preliminary clinical data that statins such as atorvastatin may exert antiepileptogenic effects in patients after stroke or TBI (Pugh et al., 2009; Etminan et al., 2010; Guo et al., 2015) and some clinical reports indicate that levetiracetam may exert such effects, as well (Jehi et al., 2012; Klein et al., 2012). Both statins and levetiracetam are multi-target drugs. Thus, in addition to their cholesterol-lowering effects, statins exert antioxidant, antiinflammatory, immunomodulatory, and antiexcitotoxic effects, which are likely to mediate antiepileptogenic efficacy (Scicchitano et al., 2015). Similarly, in addition to modulation of presynaptic neurotransmitter release via SV2A, levetiracetam exerts postsynaptic effects at GABA and glutamate receptors as well as antiinflammatory, anti-oxidative and neuroprotective effects (Löscher, 2020). However, treatment with these drugs alone is not sufficient to prevent epilepsy in the majority of patients at risk (Klein et al., 2020; Löscher, 2020).

**TABLE 4:** Antiepileptogenic and/or disease-modifying effects of selected drugs or drug combinations in chronic rat models of epilepsy and post-traumatic epilepsy prevention trials in humans. Note that, except for VX-605, all drugs shown here are in clinical use for other indications. Drug effect is indicated by + = effective; +/-, inconsistent data (+), retrospective clinical data or data from small trials; ? = no data available (or found by literature review using Pubmed). For detailed data and references see Löscher (2020) and Löscher and Klein (2021a). Abbreviations: COX, cyclooxygenase; NE, not effective; PTZ, pentylenetetrazole; TBI, traumatic brain injury; TLE, temporal lobe epilepsy.

Drug (administered *after* brain insult)	Electrical (amygdala) kindling model of TLE		Post-SE models of TLE		Post-TBI models of acquired epilepsy		Post-traumatic epilepsy in patients	
	Retardation of kindling acquisition during treatment	Disease-modification (modification of kindling upon continued stimulations after drug withdrawal)	Prevention of epilepsy	Disease-modification	Prevention of epilepsy	Disease-modification	Prevention of epilepsy	Disease-modification
Carbamazepine	NE	NE	NE	+	?	NE	NE	?
Phenytoin	NE	NE	NE	+	?	Negative effect?	NE	Negative effect?
Phenobarbital	+	+	NE	+/-	?	Negative effect?	NE	?
Valproate	+	+	NE	+	?	+	NE	NE
Levetiracetam	+	+	NE	+/-	?	+	(+)	(+)
Magnesium sulfate	?	?	?	?	?	+	NE	NE
Statins (e.g., atorvastatin)	+^a^	?	?	+	?	+	+	+
Phenytoin + phenobarbital	?	?	?	?	?	?	NE	NE
Gabapentin	?	?	?	+	?	+	?	?
COX inhibitors	+	?	+/-	+/-	?	+	?	?
Anakinra	+	?	NE^b^	+^b^	?	+	?	?
Losartan	+	?	+	+	+	+	?	?
Isofluran	?	?	+	+	?	+	?	?
Rapamycin	?	?	NE^c^	NE^c^	+	+	?	?
Anakinra and VX-605	?	?	NE	+	?	?	?	?
Levetiracetam + topiramate	?	?	+	+	?	?	?	?
Levetiracetam + topiramate + gabapentin	?	?	+	+	?	?	?	?
Levetiracetam + ceftriaxone + atorvastatin	?	?	+	+	?	?	?	?

a PTZ kindling.

b Administered together with VX-765 (a specific non-peptide inhibitor of IL-1β cleavage and release).

c When sufficiently long withdrawal after termination of treatment (see Löscher, 2020).

Because of the complexity of the processes underlying epileptogenesis, we have previously proposed that rationally chosen combinations of drugs that target multiple epileptogenic processes may be more effective to prevent or modify epilepsy than treatment with single, highly specific drugs (Löscher et al., 2013; White and Löscher, 2014; Löscher and Klein, 2021b). Clinical translation of such a network strategy would benefit from repurposing approved drugs that are currently used for other indications. In recent years, multitargeted or combinatorial therapies (“network pharmacology approaches”) have attained substantial therapeutic impact because such therapies modulate the activities of targets in complex diseases such as cancer, diabetes mellitus, hypertension, congestive heart failure, asthma, chronic obstructive pulmonary disease, and HIV-1 infection; similarly, such therapies are interesting for neurological diseases with complex etiologies unlikely to respond to single, target-specific treatments (Ainsworth, 2011; Boezio et al., 2017; Muhammad et al., 2018), such as epilepsy (Klein et al., 2018).

There are at least two principal strategies that may be used to identify an efficient network approach for the prevention or modification of epilepsy. One is the top-down approach used by Löscher’s group in the past ∼10 years, in which rationally chosen combinations of drugs that are likely to affect different targets within an epileptogenic network are tested in animal models. A second strategy is a bottom-up approach that starts by identifying crucial genomic, transcriptomic, or proteomic alterations in the network and then searches for drug targets that selectively affect these network changes (Swinney and Anthony, 2011; Eder and Herrling, 2016). The latter approach allows to develop novel, highly effective treatments, but the process of target validation is complex, long-lasting, and associated with high attrition rates (Sams-Dodd, 2005; Swinney and Anthony, 2011).

**FIGURE 8:**
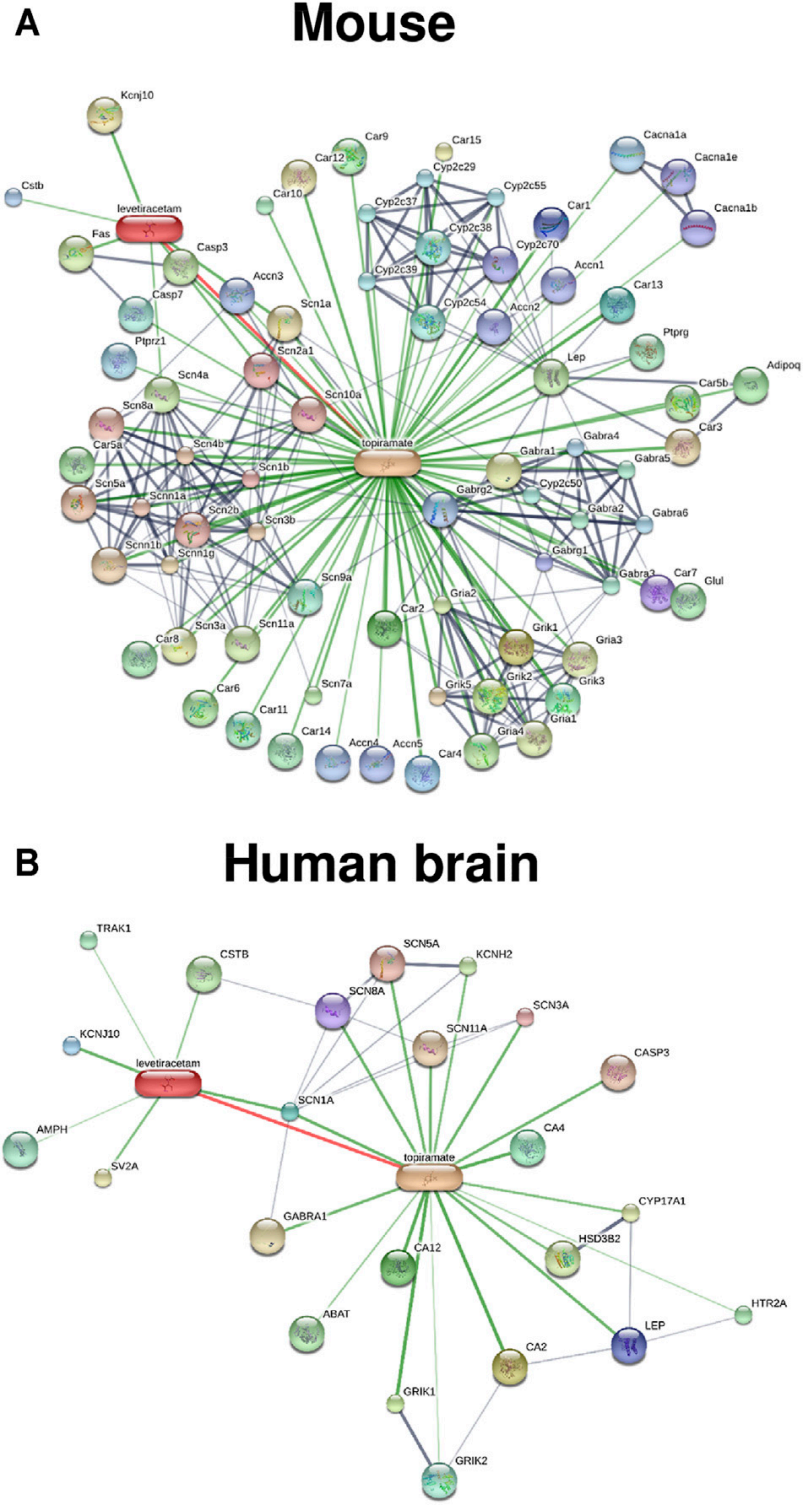
Known and predicted drug-drug protein network interactions of the combination of levetiracetam and topiramate analyzed *in silico* by the STITCH database for the mouse **(A)** and the human brain **(B)**. Tissue-specific interactions are not yet available for the mouse, explaining the higher number of interactions obtained in the mouse vs the brain of humans. Drug-protein and protein-protein networks are shown by the confidence view of the database, in which stronger associations are represented by thicker lines. Drug-protein interactions are shown in green, protein-protein interactions in grey, and interactions between drugs in red. In both species, drug interactions between levetiracetam and topiramate exist, which is indicated by the red line. Interestingly, the proteins by which the two drugs interact seems to be different in the two species (see the STITCH database (http://stitch.embl.de/) for abbreviations of proteins). However, as discussed in the text, one major disadvantage of the STITCH database is that it does not contain information on disease-associated alterations in protein networks and drug targets.

Since we proposed network pharmacology as a novel approach for epilepsy prevention or modification in 2013 (Löscher et al., 2013), we have systematically evaluated various rationally chosen combinations of repurposed drugs for tolerability and efficacy in the intrahippocampal kainate mouse model (Klee et al., 2015; Schidlitzki et al., 2017; Welzel et al., 2019; Schidlitzki et al., 2020; Welzel et al., 2021). The strategies that we used to discover effective antiepileptogenic drug combinations have been described in detail recently (Löscher and Klein, 2021b). First, a literature review of hundreds of potentially interesting clinically approved drugs was performed to select drugs with relevant MOAs and some evidence for an antiepileptogenic or disease-modifying effect in an epilepsy model. This resulted in ∼20 drugs that fulfilled all of our criteria and interacted with different processes thought to be critically involved in epileptogenesis. The next, most critical step was to decide which combinations of these drugs should be examined *in vivo*. For this purpose, we have taken two strategies: i) combining potentially synergistic drugs based on MOAs and ii) a computational *in silico* approach for network analysis, using the STITCH (Search Tool for Interacting Chemicals) database (http://stitch.embl.de/; Szklarczyk et al., 2016) to identify drug combinations that exert synergistic interactions on protein networks potentially involved in epileptogenesis (Schidlitzki et al., 2020; Welzel et al., 2021). This was followed by laborious *in vivo* studies on the antiepileptogenic potential of the 13 most promising drug combinations, using the intrahippocampal kainate mouse model (Schidlitzki et al., 2017; Schidlitzki et al., 2020; Welzel et al., 2021). The three most effective combinations resulting from *in silico* and *in vivo* evaluation are shown in Table 4: i) levetiracetam and topiramate; ii) levetiracetam, topiramate, and gabapentin, and iii) levetiracetam, ceftriaxone, and atorvastatin. *In silico* protein network analysis using the STITCH database indicated that the drugs in the three combinations are not highly selective for a single target but interact, in a complementary fashion, with various receptors and ion channels that are thought to be relevant for epileptogenesis (Schidlitzki et al., 2020; Welzel et al., 2021). An example of such an analysis for the effects of the levetiracetam plus topiramate combination in the mouse and the human brain is shown in Figure 8. Furthermore, we used gene expression analysis and multimodal brain imaging to analyze the mechanisms that underlie the synergistic efficacy of the latter drug combinations (Schidlitzki et al., 2020). Notably, only a few of the 13 drug combinations that we tested *in vivo* exhibited significant antiepileptogenic effects (Schidlitzki et al., 2017; Schidlitzki et al., 2020; Welzel et al., 2021), which in most cases was predicted by *in silico* evaluation. However, one disadvantage of *in silico* database platforms such as STITCH is that they do not contain information on disease-associated alterations in protein networks and drug targets. Thus, such platforms cannot replace the *in vivo* experiment.

To examine whether the promising drug combinations identified by our systematic approach exerted synergistic interactions *in vivo*, we determined the antiepileptogenic efficacy of monotherapy vs double or triple therapy of one of the combinations (levetiracetam, topiramate, gabapentin). Neither levetiracetam nor topiramate alone exerted any antiepileptogenic effect on electrographic or electroclinical seizures (Schidlitzki et al., 2020). Furthermore, in terms of focal electrographic (nonconvulsive) seizures, the most frequent type of SRS in the intrahippocampal kainate mouse model, only the triple combination of levetiracetam, topiramate and gabapentin significantly reduced the incidence of these seizures, while a double combination (levetiracetam, topiramate) or monotherapy were ineffective, indicating a synergistic effect of the triple combination (Löscher and Klein, 2021a; Welzel et al., 2021). Thus, these data indicate that focal electrographic (nonconvulsive) seizures are more difficult to prevent than electroclinical seizures.

We also examined the effect of different doses of the drugs in the effective combinations. First, we combined all drugs at doses that were previously reported to exert some antiepileptogenic or disease-modifying activity in other animal models of acquired epilepsy. While this resulted in antiepileptogenic efficacy of levetiracetam and topiramate as well as of levetiracetam and topiramate and gabapentin in the intrahippocampal kainate mouse model, no such efficacy was observed for the combination of levetiracetam, atorvastation and ceftriaxone (Schidlitzki et al., 2020; Welzel et al., 2021). While the antiepileptogenic effect of the levetiracetam and topiramate combination was lost when decreasing dosages by 50% (Schidlitzki et al., 2020), the opposite was found for the combination of levetiracetam, ceftriaxone, and atorvastatin, which only exerted antiepileptogenic activity when the initial dosages were decreased by 70% (Welzel et al., 2021). This illustrates the complexity of identifying antiepileptogenic drug combinations (Löscher and Klein, 2021b).

In addition to our systematic approach, several other groups evaluated drug combinations for antiepileptogenic efficacy. An example, in which anakinra, a recombinant version of the human interleukin 1 (IL) receptor antagonist, and VX-605, a specific non-peptide inhibitor of IL-1β cleavage and release, were combined (Noé et al., 2013) is shown in Table 4. The relative moderate effects of this combination may indicate that combining two compounds that act on similar targets (neuroinflammation in this example) is less effective than combining compounds that act on different targets. However, a more recent study in which two antioxidant compounds (4-(2-aminoethyl)-benzenesulfonyl fluoride (AEBSF) and RTA 408) were combined resulted in a marked (67%) decrease in SRS incidence in the kainate model in rats (Shekh-Ahmad et al., 2019). As in our study with topiramate and levetiracetam (Schidlitzki et al., 2020), the combination was more effective than either drug alone (Shekh-Ahmad et al., 2019). However, in another study in which the two antioxidant drugs N-acetylcysteine and sulforaphane were combined, no significant effect on SRS incidence was found in an electrically-induced SE model of TLE in rats (Pauletti et al., 2019).

Concerning the effect of DMLs on epileptogenesis, it would be interesting to know whether and how padsevonil interferes with the development of epilepsy after brain injury. Recently, brivaracetam has been found to exert antiepileptogenic effects in a rat model of PTE (Eastman et al., 2021). Thus, one would expect that padsevonil is more effective than the single-target drug brivaracetam in preventing the development of epilepsy in a relevant model of acquired disease.

## Conclusion

Although the development of DMLs for the treatment of epilepsy was proposed 27 years ago (Löscher and Schmidt, 1994), the implementation and validation of this strategy is still in its infancy. Indeed, to my knowledge, padsevonil is the only rationally designed multimodal drug that has been developed for epilepsy therapy and has undergone clinical trials. The fact that padsevonil failed in a randomized placebo-controlled phase IIb add-on trial in patients with treatment-resistant focal epilepsy (French, 2020a) does not mean that this drug is not exerting antiseizure efficacy in patients with less severe types of epilepsy. However, UCB's goal of padsenonil becoming a game-changer for the population with the greatest unmet need in epilepsy was not fulfilled. In contrast, as described in *Padsevonil, the First Rationally Designed Multimodal Antiseizure Medication*, the novel multimodal ASM cenobamate brings substantial promise for patients with ASM-resistant seizures, although this drug was not developed by any rational, target-based strategy (French, 2020b). This illustrates a dilemma in ASM development (Löscher and Schmidt, 2011), i.e., that the best strategy to find a magic bullet for epilepsy therapy is still unknown. Nonetheless, it is clear that phenotypic screening, chemocentric approaches, and serendipity remain to be more effective strategies than target-based drug development. This disappointing fact is not restricted to ASMs but is also true in many other fields of drug development (Swinney and Anthony, 2011; Baumeister et al., 2013; Swinney, 2013; Eder and Herrling, 2016; Croston, 2017), although one study came to different conclusions as to which target-based strategy is more successful in discovering first-in-class medicines (Eder et al., 2014). In recent years, there has been a resurgence in interest in phenotypic drug screening approaches based on their potential to address the incompletely understood complexity of diseases and their promise of delivering first-in-class drugs, as well as major advances in the tools for cell-based phenotypic screening (Haasen et al., 2017; Moffat et al., 2017). For complex diseases such as epilepsy, the high attrition rates of drug candidates in clinical trials could partly result from an underestimation of the complexity of the pathophysiology in these diseases (Gintant and George, 2018). As described in *Preclinical Discovery and Development of Antiseizure Medications* and *Single-Target Rather Than Multi-Target Drugs for Genetic Epilepsies: The Development of Precision Medicines*, drug repurposing has emerged as a viable strategy to increase the overall productivity of drug discovery (Cha et al., 2018). Unbiased, high-throughput screens can be used systematically in combination with bioinformatics to test libraries of clinically approved drugs against the disease process in areas of medicine outside of their usual indications (Massey and Robertson, 2018). Furthermore, in principle, rationally designed multimodal drugs (DMLs) should have advantages for the treatment of a complex disease such as epilepsy, provided that the mechanisms underlying ASM-resistant seizures are sufficiently understood (Löscher et al., 2020). In fact, insufficient understanding of disease and treatment mechanisms is a major barrier in DML development (Bain et al., 2017; Ramsay et al., 2018). Furthermore, the complexity of the brain and our poor understanding of neurological disease mechanisms in general makes DML development demanding. Also, for epilepsy therapy, it remains to be shown that a DML provides advantages in antiseizure efficacy vs add-on or monotherapy with approved ASMs or combinations of ASMs with different MOAs. In the near future, the striking advances in the “omics”, *in silico* (computational) approaches, including data mining, mathematical modeling, information visualization methods, as well as machine learning approaches will likely make a fundamental impact on the speed and accuracy of predictions during the process of drug development and hopefully lead to more effective multimodal drugs both for the therapy and prevention of epilepsy.

## Abbreviations

AMPA, α-amino-3-hydroxy-5-methyl-4-isoxazolepropionic acid; ASM, antiseizure medication; ASP, Anticonvulsant Screening Program; CD97, convulsant dose in 97% of the animals; CLN2, ceroid lipofuscinosis type 2; DML, designed multiple ligand; ED50, effective dose suppressing seizures in 50% of the animals; ETSP, Epilepsy Therapy Screening Program; GABA, γ-aminobutyric acid; GAERS, Genetic Absence Epilepsy Rat from Strabourg; GAT, GABA transporter; IL, interleukin; MES, maximal electroshock seizure; MOA, mechanism of action; mTLE, mesial temporal lobe epilepsy; NCE, new chemical entity; NINDS, National Institutes of Neurological Disorders and Stroke; NMDA, N-methyl-d-aspartate; PET, positron emission tomography; PAM, positive allosteric modulator; PTZ, pentylenetetrazole; SE, status epilepticus; SEGA, subependymal giant cell astrocytoma; SRS, spontaneous recurrent seizures; STITCH, Search Tool for Interacting Chemicals; SV2A, synaptic vesicle protein 2A; TBI, traumatic brain injury; TSC, tuberous sclerosis complex; WAG/Rij, Wistar Albino Glaxo from Rijswijk.
